# Nutrition and Vitamin Deficiencies Are Common in Orthopaedic Trauma Patients

**DOI:** 10.3390/jcm10215012

**Published:** 2021-10-28

**Authors:** Jordan E. Handcox, Jose M. Gutierrez-Naranjo, Luis M. Salazar, Travis S. Bullock, Leah P. Griffin, Boris A. Zelle

**Affiliations:** 1 Department of Orthopaedics, UT Health San Antonio, 7703 Floyd Curl Dr., San Antonio, TX 78229, USA; handcox@uthscsa.edu (J.E.H.); gutierreznar@uthscsa.edu (J.M.G.-N.); bullockt@uthscsa.edu (T.S.B.); 2 Long School of Medicine, UT Health San Antonio, 7703 Floyd Curl Dr., San Antonio, TX 78229, USA; salazarlm@livemail.uthscsa.edu; 3 Medical Solutions Division, 3M Health Care, San Antonio, TX 78249, USA; lpgriffin@mmm.com

**Keywords:** orthopaedic trauma, nutritional deficiencies, vitamins, lower extremity, wound complications, nutrition wound healing

## Abstract

Macro- and micronutrients play important roles in the biological wound-healing pathway. Although deficiencies may potentially affect orthopaedic trauma patient outcomes, data on nutritional deficiencies in orthopaedic trauma patients remain limited in the literature. The purpose of this study was to (1) evaluate the prevalence of macro- and micronutrient deficiencies in orthopaedic trauma patients with lower extremity fractures and (2) evaluate the impact of such deficiencies on surgical site complications. This retrospective study identified 867 patients with lower extremity fractures treated with surgical fixation from 2019 to 2020. Data recorded included albumin, prealbumin, protein, vitamins A/C/D, magnesium, phosphorus, transferrin and zinc, as well as wound complications. Nutritional deficiencies were found for prealbumin, albumin and transferrin at 50.5%, 23.4% and 48.5%, respectively. Furthermore, a high prevalence of micronutrient deficiencies (vitamin A, 35.4%; vitamin C, 54.4%; vitamin D, 75.4%; and zinc, 56.5%) was observed. We also recorded a statistically significant difference in wound complications in patients who were deficient in prealbumin (21.6% vs. 6.6%, *p* = 0.0142) and vitamin C (56.8% vs. 28.6%, *p* = 0.0236). Our study outlines the prevalence of nutritional deficiencies in an orthopaedic trauma population and identifies areas for possible targeted supplementation to decrease wound complications.

## 1. Introduction

The important role of nutrition in wound healing has been well documented in the literature, with macro- and micronutrients considered vital at every step of the wound healing pathway [1,2,3]. Unfortunately, malnutrition is common worldwide [4] and can be from a variety of causes, including advanced age, disease-related, food-insecurity/hunger or a mismatch between caloric intake and quality of nutrients consumed [5]. Malnutrition is a known contributor to poor clinical outcomes, from increased morbidity and mortality to wound and surgical complications [6,7]. As such, there is much interest in evaluating the role of nutrition in orthopaedic trauma patients, a vulnerable population sensitive to the effects of malnutrition.

Previous literature has demonstrated that malnutrition, as defined by hypoalbuminemia, is common in the orthopaedic trauma population [8]. Moreover, these authors recorded hypoalbuminemia and obesity as predictors of wound complications. Additionally, albumin deficiencies have been shown to correlate with wound complications in patients undergoing joint replacement surgery [9] and readmission rates for patients undergoing elective spine surgeries [10]. However, prior research has mostly focused on the geriatric populations, elective orthopaedic surgeries, and albumin and prealbumin as serum markers for malnutrition [11,12,13,14].

Even fewer studies have looked at the prevalence of micronutrient deficiencies in orthopaedic trauma patients. Among elderly hospitalized patients, vitamins C and D are commonly deficient [15,16]; however, there is limited data on its prevalence among a younger trauma population. Severe micronutrient deficiencies have well-known consequences, such as severe vitamin D deficiency leading to rickets and osteoporosis. Subtle deficiencies below the reference range may lead to wound-healing complications and other lesser-known sequela [5], and the orthopaedic literature has just started to explore the relationship between micronutrient deficiencies and negative clinical outcomes. For example, it has been shown that zinc deficiencies may lead to wound-healing complications in patients undergoing hemiarthroplasty [17]. Also, vitamin D has been shown to impact fracture healing rates in orthopaedic trauma patients [18]. Yet, data on micronutrient deficiencies in the orthopaedic trauma population remain limited in the literature.

The purpose of this study is to (1) evaluate the prevalence of macro- and micronutrient deficiencies in orthopaedic trauma patients with lower extremity fractures and (2) evaluate the impact of such deficiencies on surgical site complications in patients with high-risk lower extremity fractures. Our hypothesis is that deficiencies are common in the orthopaedic trauma population, and these deficiencies may be associated with an increase in surgical site complications in high-risk lower extremity fractures.

## 2. Materials and Methods

This study is a retrospective database analysis of orthopaedic trauma patients undergoing surgical fixation of their lower extremity fractures treated at a university-based level 1 trauma center between the years of 2019 and 2020. The study protocol was approved by our Institutional Review Board, and data collection, methods and analysis were performed in accordance with their rules and regulations. Inclusion criteria were patients over 18 years old with a minimum of 3 months follow-up and lower extremity fractures, identified through our electronic medical record system using the coding database. Patients were identified using the OTA classification system to include femur, tibia, tibia/fibula, fibula, talus, calcaneus and foot fractures. Subjects were excluded if they were under 18 years old, mentally or cognitively impaired, prisoners, or if they presented with a pathologic fracture, as well as those with less than 3 months follow-up.

Demographic data included age, gender, race/ethnicity, BMI, and the American Society of Anesthesiologists scale [19], as well as mechanism of injury and closed versus open injury. As our primary outcome measure, we recorded the available laboratory data on patients, including both macro- and micronutrient data: albumin (3.2–5.0 g/dL), prealbumin (17.0–37.1 mg/dL), protein total serum (6.2–8.1 g/dL), albumin/globulin ratio (1.06–1.61), transferrin (206–382 mg/dL), vitamin A (0.30–1.20 mg/L), vitamin C (23–114 mmol/L), vitamin D (30–80 ng/mL), vitamin K (0.22–4.88 nmol/L), magnesium (1.6–2.2 mg/dL), phosphorus (2.4/4.6 mg/dL), zinc (60.0–120 µg/dL), selenium (23–190 µg/L), TSH (0.350–5.500 µIU/mL) and PTH (19–88 pg/mL). An expanded panel of micronutrient data was obtained for patients who were deemed high risk by their treating orthopaedic surgeon; this expanded lab draw was at the discretion of the surgeon.

Secondary clinical outcome measures were tracked through a review of inpatient and outpatient charts and included data on wound complications (surgical site infections, wound dehiscence, hematoma) and surgical complications (malunion, nonunion, symptomatic hardware).

The statistical analysis was performed using SAS software version 9.4 (SAS Institute Inc., Cary, NC, USA). Categorical variables are summarized as count and percent. Chi-squared and Fisher’s exact test were used to calculate differences for categorical variables. All tests were conducted at the alpha level of 0.05.

## 3. Results

### 3.1. Patient-Level Demographic Data

We identified 867 patients who met the inclusion criteria. Of these patients, 28.7% were age 65 or older. There were slightly more male patients (56.9%) compared to female patients. A majority of patients identified as White (95.6%) or Hispanic/Latino (62.1%). Finally, nearly 40% of patients were obese, as defined by BMI ≥ 30 (Table 1).

### 3.2. Nutritional Deficiencies

Albumin was measured for 745 patients, and of these, 23.4% were malnourished, as defined by albumin < 3.5 g/dL. Approximately half of the patients were deficient in prealbumin (50.5%). Finally, nearly half of the patients (48.5%) were deficient in transferrin.

Of those patients who had micronutrient data measured, 35.4% were deficient in vitamin A, 54.4% were deficient in vitamin C, and 75% were deficient in vitamin D. Over half of the patients were deficient in zinc (56.5% of patients). We did not observe significant deficiencies in magnesium, selenium or vitamin K (Table 2).

### 3.3. Nutritional Deficiencies by Demographic Group

There were significant differences in nutritional deficiencies between demographic groups. Of the patients 65 and older, 83.3% were deficient in prealbumin, compared to only 46.0% of patients younger than 65 years old (*p* = 0.0153). A similar trend was recorded with age and albumin, where 37% of patients over 65 years old were deficient, compared to 17.7% of patients under 65 years old (*p* < 0.0001). Compared to younger patients, patients over 65 years old were also at increased risk of deficiency in serum protein (11.7% deficient versus 7.1%, *p* = 0.0396) and transferrin (81.8% deficient versus 42.1%, *p* = 0.0158). With regards to age-related differences in micronutrients, patients over 65 years old were not at increased risk of deficiency in vitamins A, C, or zinc. Additionally, advanced age was protective against vitamin D deficiency, as 84.7% of younger patients had vitamin D deficiency, compared to 59.0% of patients over 65 (*p* < 0.0001).

Females were more likely to be deficient in albumin (29.2%) compared to males (19.2%, *p* = 0.0015). Micronutrient data showed that Hispanic patients were more likely to be vitamin D deficient than non-Hispanic patients (82.4% versus 65.6%, *p* = 0.0047). We did not observe statistically significant differences between these demographic groups in the remaining serum markers.

### 3.4. Complications by Nutritional Deficiency

To measure the rate of wound complications, we assessed the data at the fracture level, identifying 1008 individual lower extremity fractures, 181 (18.0%) of which had a wound complication. Low prealbumin was associated with a statistically significant difference in wound complications. We found that 21.6% of fractures with a prealbumin deficiency had a wound complication, compared to 6.6% of those with normal prealbumin levels (*p* = 0.0142). Vitamin C deficiency was also associated with wound complications; where 56.8% sustained a wound complication, compared to only 28.6% with normal vitamin C levels (*p* = 0.0236).

## 4. Discussion

Identifying macro- and micronutrient deficiencies in our orthopaedic trauma population is important, as it allows us to (1) understand the prevalence of this issue and (2) perform targeted interventions, which may lead to improvement in outcomes. Our data suggest that nutritional deficiencies have a high prevalence in orthopaedic trauma patients. Furthermore, macro- and micronutrient deficiencies may be associated with wound complications, most notably prealbumin and vitamin C deficiency.

There were some limitations to our study. One limitation was its retrospective design; this resulted in limited data for some of the studied micronutrients. Also, expanded micronutrient data collection was at the discretion of the treating surgeon at the time of hospital presentation. This led to variability in which nutritional markers were drawn for each patient. This may have contributed to the underrepresentation of certain micronutrients and difficulties in identifying other surgical complications, such as malunion/nonunion. Also, these data are from our local orthopaedic trauma population, which may lead to geographic variation in deficiency patterns.

Previous studies demonstrated improved outcomes with nutritional supplements in the geriatric population [14,20] and enhanced callus formation with zinc supplementation in young adult trauma patients with lower extremity fractures [21]. To our knowledge, there have not been any data reported on nutritional deficiencies using multiple serum markers in orthopaedic trauma patients with injuries at high risk for infection.

We have demonstrated profound malnutrition rates, including a hypoalbuminemia rate of 23.4%, which is slightly lower than previously found in this population (39.4%, Egbert et al.). Over half of our patients were deficient in prealbumin, which more closely correlates with perioperative nutritional deficiency, given its shorter half-life [22]. Prealbumin deficiency has previously been shown to correlate with surgical site infections in patients undergoing spinal surgery [23], and we did confirm a statistically significant difference in wound complications among those who were prealbumin deficient in our population. Almost half of the patients (48.5%) were deficient in transferrin, which also indicates significant malnutrition [24] and has been implicated in wound complications in arthroplasty [25]. Regarding micronutrient deficiencies, we found substantial deficiencies in vitamins A, C, D, and zinc, which is in line with the data available in the geriatric population [11,14]. In addition, Hispanic patients were also much more likely to be vitamin D deficient. Finally, we demonstrated that vitamin C deficiency is common, as is consistent with the existing literature [15] and found that deficiency in vitamin C may lead to wound complications.

Our study reinforces prior literature on the prevalence and impact of hypoalbuminemia in an orthopaedic trauma population [8,26] and confirms that malnutrition is a risk factor for wound complications [27,28]. Our study also confirms prior research demonstrating significant vitamin D deficiency in a diverse trauma population [12]. Our novel data on micronutrient deficiencies in the orthopaedic trauma population provides preliminary evidence for vitamin/nutritional supplementation in the perioperative period for a younger orthopaedic trauma population in order to improve clinical outcomes.

## 5. Conclusions

In summary, our study demonstrates a high prevalence of macro- and micronutrient deficiencies in an orthopaedic trauma patient population with lower extremity fractures. Deficiencies in prealbumin, and vitamins C, D and zinc were common, with over half of patients in our study group proving to be deficient. We also identified demographic risk factors for malnutrition, such as age, sex and ethnicity. Finally, we demonstrated that prealbumin and vitamin C may be associated with wound complications. This study lays the groundwork for identifying targeted supplement and nutritional interventions that may reduce the risk of surgical site complications. The reversal of these deficiencies in the perioperative period has the potential to improve patient outcomes and reduce hospital costs.

## Figures and Tables

**Table 1:** Demographic and clinical data.

Number of Patients	*n* = 867
Age ≥ 65	249 (28.7%)
Ethnicity—Hispanic	538 (62.1%)
Race—White	829 (95.6%)
Gender (% Male)	493 (56.9%)
BMI ≥ 30	343 (39.6%)
Fracture location	*n* = 1008
Proximal Femur	195 (19.4%)
Femoral Shaft	107 (10.6%)
Distal Femur	34 (3.4%)
Proximal Tibia	98 (9.7%)
Tibial Shaft	84 (8.3%)
Ankle/Pilon	352 (34.9%)
Talus	20 (2.0%)
Calcaneus	51 (5.1%)
Foot	17 (1.7%)
Infection, Non-traumatic	38 (3.8%)
Others	12 (1.2%)

**Table 2:** Nutritional deficiencies by macro- or micronutrient.

Nutritional Markers	*N*	Deficient
Prealbumin	99	50 (50.5%)
Albumin	745	174 (23.4%)
Protein total serum	735	62 (8.4%)
Albumin/globulin ratio	734	512 (69.8%)
Transferrin	68	33 (48.5%)
Vitamin A	82	29 (35.4%)
Vitamin C	57	31 (54.4%)
Vitamin D	215	162 (75.4%)
Vitamin K	42	1 (2.4%)
Magnesium	619	21 (3.4%)
Phosphorus	609	100 (16.4%)
Zinc	92	52 (56.5%)
Selenium	63	0 (0%)
Thyroid-stimulating hormone	154	9 (5.8%)
Parathyroid hormone	97	2 (2.1%)

## Data Availability

The data are not publicly available online.
